# Early peritoneal metastasis after laparoscopic incisional hernia repair secondary to colon cancer resection: A case report

**DOI:** 10.1016/j.amsu.2021.103000

**Published:** 2021-10-30

**Authors:** Erika Machida, Shingo Tsujinaka, Nao Kakizawa, Yasuyuki Miyakura, Koichi Suzuki, Toshiki Rikiyama

**Affiliations:** Department of Surgery, Saitama Medical Center, Jichi Medical University, Affiliation Address: 1-847, Amanumacho, Omiya, Saitama-shi, Saitama, 330-8503, Japan

**Keywords:** Peritoneal metastasis, Incisional hernia, Colon cancer

## Abstract

**Introduction:**

and importance: We report a case of a patient who developed early peritoneal metastasis after laparoscopic incisional hernia repair secondary to curative colon cancer resection.

**Case presentation:**

A 77-year-old woman underwent ileocecal resection with open laparotomy for locally advanced cecal cancer. The pathological diagnosis was adenocarcinoma with T3N2aM0. Three months after the surgery, she developed incisional hernia at the midline incision site. After the completion of adjuvant chemotherapy, surveillance computed tomography (CT) showed no cancer recurrence. Her abdominal discomfort persisted because of incisional hernia, and thus we performed laparoscopic incisional hernia repair using the intraperitoneal onlay mesh technique at 11 months after the initial surgery.

Five months after incisional hernia repair, CT showed multiple liver and peritoneal metastases. She was started on systemic chemotherapy. Two days after the first therapeutic infusion, she developed small bowel obstruction. We decided to perform palliative surgery with intestinal bypass. Exploratory laparoscopy revealed that the implanted mesh for incisional hernia repair was completely covered with multiple nodules of peritoneal metastasis. Two months after the bypass surgery, she resumed her chemotherapy, but CT showed significant progression of all recurrent lesions. She did not wish to continue further chemotherapy and decided to receive the best supportive care.

**Clinical discussion:**

This case may raise important clinical questions regarding the indication and timing of incisional hernia repair for patients who are at high risk of cancer recurrence.

**Conclusion:**

Incisional hernia repair must be performed in the absence of any possibility of cancer recurrence, particularly in the earlier follow-up period.

## Highlights

• We present a case of peritoneal metastasis of colon cancer after incisional hernia repair.
• Incisional hernia repair using mesh was performed after completion of adjuvant chemotherapy.
• Peritoneal metastasis occurred 5 months after laparoscopic incisional hernia repair.
• The indication and timing of incisional hernia repair should be carefully considered.
• This case may raise the question on the relationship between the mesh and peritoneal metastasis.

## 1 Introduction and importance

Incisional hernia after colorectal cancer resection reportedly occurs in 7.0%–8.5% of postoperative patients [1,2]. However, the surgical indication and optimal timing of its repair have not been elucidated, particularly for the patients who are at high risk of cancer recurrence (e.g., peritoneal metastasis).

We herein report a case of a patient who developed early metachronous peritoneal metastasis after laparoscopic incisional hernia repair secondary to curative colon cancer resection. This study has been reported in line with the SCARE criteria [3].

## 2 Case presentation

A 77-year-old woman presented with weight loss and pain in the right lower abdomen that started 1 month prior to admission. Her past medical history was significant for hypertension requiring medication. Her family history did not include any colorectal cancer. Laboratory data showed anemia and an elevated carcinoembryonic antigen (CEA) level of 5.7 ng/mL. Colonoscopy showed an obstructive, advanced tumor in the cecum, and biopsy revealed adenocarcinoma. Abdominal computed tomography (CT) showed an irregular, contrast-enhanced wall thickening in the cecum with enlarged pericolic lymph nodes. She was diagnosed with locally advanced cecal cancer. Considering the progressive pain and small bowel dilation caused by the obstructive large tumor, we performed ileocecal resection with open laparotomy. The pathological diagnosis was tubular and mucinous adenocarcinoma with T3N2aM0 (stage IIIB, UICC TNM classification 8th edition [4]). Postoperative course was uneventful, and the CEA level was subsequently normalized.

**Fig. 1:**
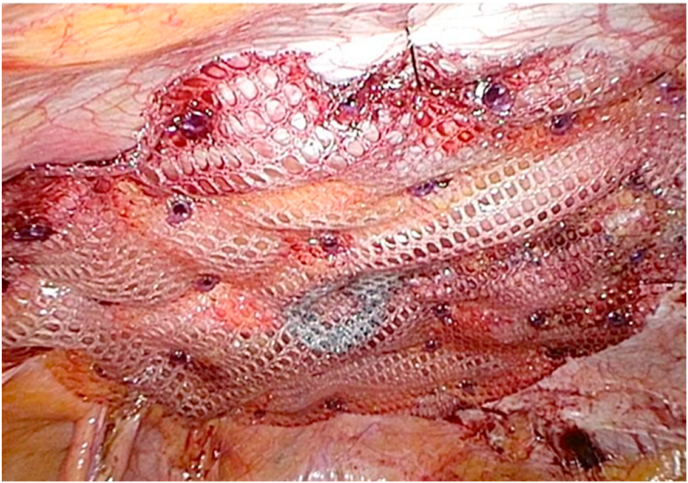
Intraoperative image of laparoscopic incisional hernia repair using a mesh.

She was started on capecitabine plus oxaliplatin (CAPOX: oral capecitabine 2000 mg/m^2^ daily on days 1–14 plus intravenous oxaliplatin 130 mg/m^2^ on day 1 of a 3-week cycle) therapy as adjuvant chemotherapy from 6 weeks after the surgery. Three months after the surgery, she noticed abdominal bulging at the midline incision site. She was diagnosed with incisional hernia on physical examination. She had tolerated 8 courses of CAPOX therapy. After the completion of adjuvant chemotherapy, surveillance CT was performed and showed no cancer recurrence (10 months after the surgery). Her abdominal discomfort persisted because of incisional hernia, and she claimed that her daily quality of life (QOL) had been deteriorated. She wished to undergo surgical intervention for incisional hernia. Therefore, we planned of laparoscopic incisional hernia repair 11 months after the initial surgery. Laparoscopic repair was performed using the intraperitoneal onlay mesh technique. Exploratory laparoscopy showed no liver or peritoneal metastasis. The hernia orifice was 5.2 cm × 5.0 cm in size with minimal adhesions. The defect was closed using absorbable barbed suture, and a multifilament polyester mesh with a bioabsorbable collagen film was placed to cover the defect. The mesh was trimmed to obtain a 5-cm overlap for the defect. The mesh was fixed with prefixed threads and absorbable tacks by the double-crown technique (Fig. 1).

**Fig. 2:**
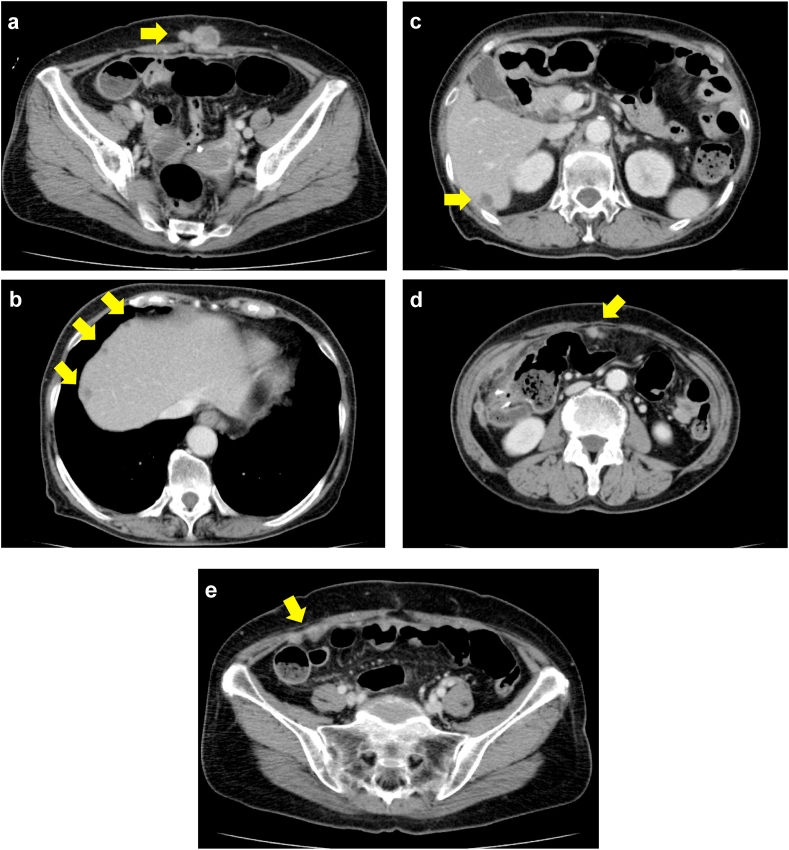
Computed tomography findings of cancer recurrence. a: abdominal wall metastases, b, c: multiple liver metastases, d, e: multiple peritoneal metastases.

Five months after incisional hernia repair (16 months after the initial surgery), surveillance CT showed abdominal wall metastases in the midline and multiple liver and peritoneal metastases (Fig. 2a–e). The CEA level increased to 9.9 ng/mL. These metastatic lesions were obviously unresectable, indicating systemic chemotherapy. Because the previously resected colon cancer specimen showed RAS mutation, we selected FOLFIRI plus bevacizumab regimen consisted of bevacizumab (5 mg/kg), irinotecan (150 mg/m^2^), bolus FU (400 mg/m^2^) and leucovorin (400 mg/m^2^), followed by 46-h FU infusions (2400 mg/m^2^). Two days after the first therapeutic infusion, she had nausea and vomiting. Abdominal CT showed small bowel obstruction. Conservative treatment was initiated with fasting and intestinal intubation, but her obstructive symptoms had repeatedly occurred at short intervals. Thereafter, we decided to perform palliative surgery with intestinal bypass.

**Fig. 3:**
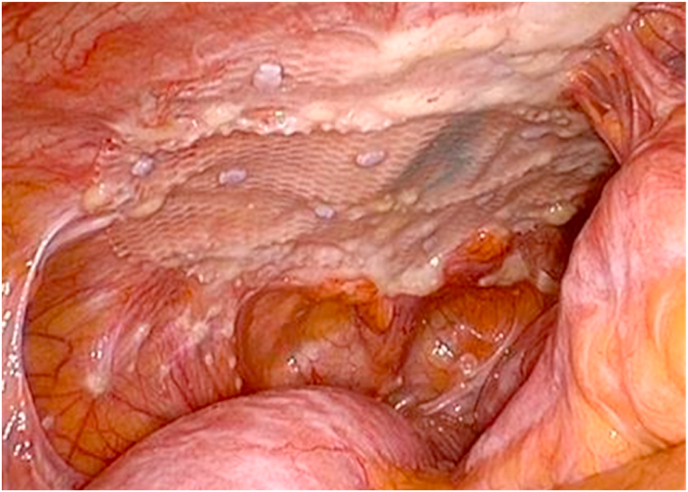
Intraoperative image at the time of intestinal bypass. The implanted mesh for incisional hernia repair is completely covered with multiple nodules of peritoneal metastasis.

The surgery was commenced with exploratory laparoscopy, which revealed local recurrence around the anastomosis and that the mesh used for incisional hernia repair was completely covered with multiple nodules of peritoneal metastasis (Fig. 3). Then, we performed laparotomy in the left upper quadrant and constructed intestinal bypass between the jejunum and transverse colon. Two months after the bypass surgery, she resumed on FOLFIRI plus bevacizumab regimen (the same as the aforementioned protocol). After 4 courses, CT showed significant progression of all recurrent lesions. At this point, she did not wish to undergo further chemotherapy and decided to receive the best supportive care. She was transferred to a nursing facility and died 2 years after the initial surgery.

## 3 Clinical discussion

The peritoneal recurrence of colorectal cancer occurs in 3.5%–5.1% of patients who underwent radical resection [5,6]. The risk factors for metachronous peritoneal metastasis are as follows: advanced tumor stage (stage II or III), T4 cancer, lymph node positivity, mucinous adenocarcinoma, macroscopic growth features (infiltrating or ulcero-infiltrating type), positive or unknown resection margin, history of perforation or obstruction, and tumor location (colon cancer) [[5], [6], [7]]. The median time to diagnosis of peritoneal recurrence is 16–18 months after the initial surgery [6,7].

The patient had advanced colon cancer with lymph node metastasis (T3N2aM0), which corresponded to the risk factor for peritoneal recurrence. Moreover, the diagnosis of peritoneal recurrence was made 16 months after the initial surgery, which is similar to that of the recent literature [6,7]. On the other hand, the median time from colorectal cancer resection to incisional hernia repair has been reportedly 1.4–1.6 years [8,9]. In this case, incisional hernia repair was performed at 11 months after the initial surgery, which was slightly earlier than those in the literature.

Incisional hernia secondary to colon cancer resection influences patients’ QOL. These patients experience pain, dyspnea, and insomnia and have decreased global health and physical, role, emotional, and social functioning in the European Organisation for Research and Treatment of Cancer QOL scores [9]. The patient also had abdominal discomfort with deteriorated QOL and thus wished to have surgical intervention for incisional hernia.

In this case, we performed laparoscopic incisional hernia repair after adjuvant chemotherapy at 11 months after the initial surgery. She was diagnosed with stage IIIB colon cancer, which carried a high risk of recurrence. Microscopic dissemination may occur before or during surgery and macroscopic dissemination may be visible after a certain period of time after surgery. Therefore, it may have been optional to plan incisional hernia repair later than 16 months after the initial surgery, which was regarded as the median time to peritoneal recurrence [6,7]. Furthermore, she had not only peritoneal and abdominal wall recurrences but also local recurrence and liver metastasis at 5 months after incisional hernia repair. We assume that the cause of cancer recurrence was highly attributed to the oncologic behavior rather than the incisional hernia repair using a mesh.

We believe that incisional hernia repair for this case may be justified in improving her QOL. However, exploratory laparoscopy during the last surgery showed multiple abdominal wall metastases where the hernia defect was closed and multiple peritoneal metastases to the surfaces of the mesh and tackers (Fig. 3). These findings raise important clinical questions regarding the indication and timing of incisional hernia repair for patients who are at high risk of cancer recurrence. To our knowledge, there has been no evidence that the mesh used for incisional hernia repair directly triggers peritoneal metastasis. However, some recent preclinical studies have shown that intraperitoneal mesh induced the deposition of neoperitoneum and tissue ingrowth [10,11]. Furthermore, mesothelial cells identified on the implanted mesh may be derived from free-floating precursor cells located in the peritoneal cavity [12]. Therefore, it is interesting to investigate the relationship between the mesh and metachronous peritoneal metastasis for further research.

## 4 Conclusion

Incisional hernia secondary to colon cancer resection may cause decreased patients' QOL if symptomatic. The indication and timing of incisional hernia repair can be determined based on the patients’ request and their symptoms. Furthermore, the surgery must be performed without any possibility of cancer recurrence, particularly in the earlier follow-up period.

## Declarations of competing interest

All authors declare that there are no competing interests.

## Sources of funding

This research did not receive any specific grant from any funding agency in the public, commercial, or not-for-profit sectors.

## Ethics approval

The institutional ethics committee (Bioethics Committee for Clinical Research, Saitama Medical Center, Jichi Medical University) determined that approval was not necessary for a case report.

## Consent

Written informed consent was obtained from the patient for publication of this case report and any accompanying images. A copy of the written consent is available for review by the Editor-in-Chief of this journal on request.

## Author contributions

Conceptualization: Erika Machida, Shingo Tsujinaka. Investigation: Erika Machida, Shingo Tsujinaka. Data curation and visualization: Nao Kakizawa. Supervision: Yasuyuki Miyakura, Koichi Suzuki. Project administration: Toshiki Rikiyama. Writing-original draft: Erika Machida, Shingo Tsujinaka. Writing-review and editing: Yasuyuki Miyakura, Koichi Suzuki, Toshiki Rikiyama. All authors have read and approved the final manuscript for submission.

## Guarantor

Shingo Tsujinaka, the corresponding author of this paper.

## Research registration

Not applicable.

## Provenance and peer review

Not commissioned, externally peer-reviewed.

## Annals of medicine and surgery

The following information is required for submission. Please note that failure to respond to these questions/statements will mean your submission will be returned. If you have nothing to declare in any of these categories, then this should be stated.

## Please state any conflicts of interest

All authors must disclose any financial and personal relationships with other people or organisations that could inappropriately influence (bias) their work. Examples of potential conflicts of interest include employment, consultancies, stock ownership, honoraria, paid expert testimony, patent applications/registrations, and grants or other funding.

All authors declare that there is no conflict of interest.
